# Fusion Genes Altered in Adult Malignant Gliomas

**DOI:** 10.3389/fneur.2021.715206

**Published:** 2021-10-04

**Authors:** Gan You, Xing Fan, Huimin Hu, Tao Jiang, Clark C. Chen

**Affiliations:** ^1^Department of Neurosurgery, Beijing Tiantan Hospital, Capital Medical University, Beijing, China; ^2^Department of Neurophysiology, Beijing Neurosurgical Institute, Beijing, China; ^3^Department of Molecular Pathology, Beijing Neurosurgical Institute, Beijing, China; ^4^Department of Neurosurgery, University of Minnesota, Minneapolis, MN, United States

**Keywords:** fusion gene, high-grade glioma, glioblastoma, targeted therapy, personalized cancer medicine

## Abstract

Malignant gliomas are highly heterogeneous brain tumors in molecular genetic background. Despite the many recent advances in the understanding of this disease, patients with adult high-grade gliomas retain a notoriously poor prognosis. Fusions involving oncogenes have been reported in gliomas and may serve as novel therapeutic targets to date. Understanding the gene fusions and how they regulate oncogenesis and malignant progression will contribute to explore new approaches for personalized treatment. By now, studies on gene fusions in gliomas remain limited. However, some current clinical trials targeting fusion genes have presented exciting preliminary findings. The aim of this review is to summarize all the reported fusion genes in high-grade gliomas so far, discuss the characterization of some of the most popular gene fusions occurring in malignant gliomas, as well as their function in tumorigenesis, and the underlying clinical implication as therapeutic targets.

## Background

**Figure 1:**
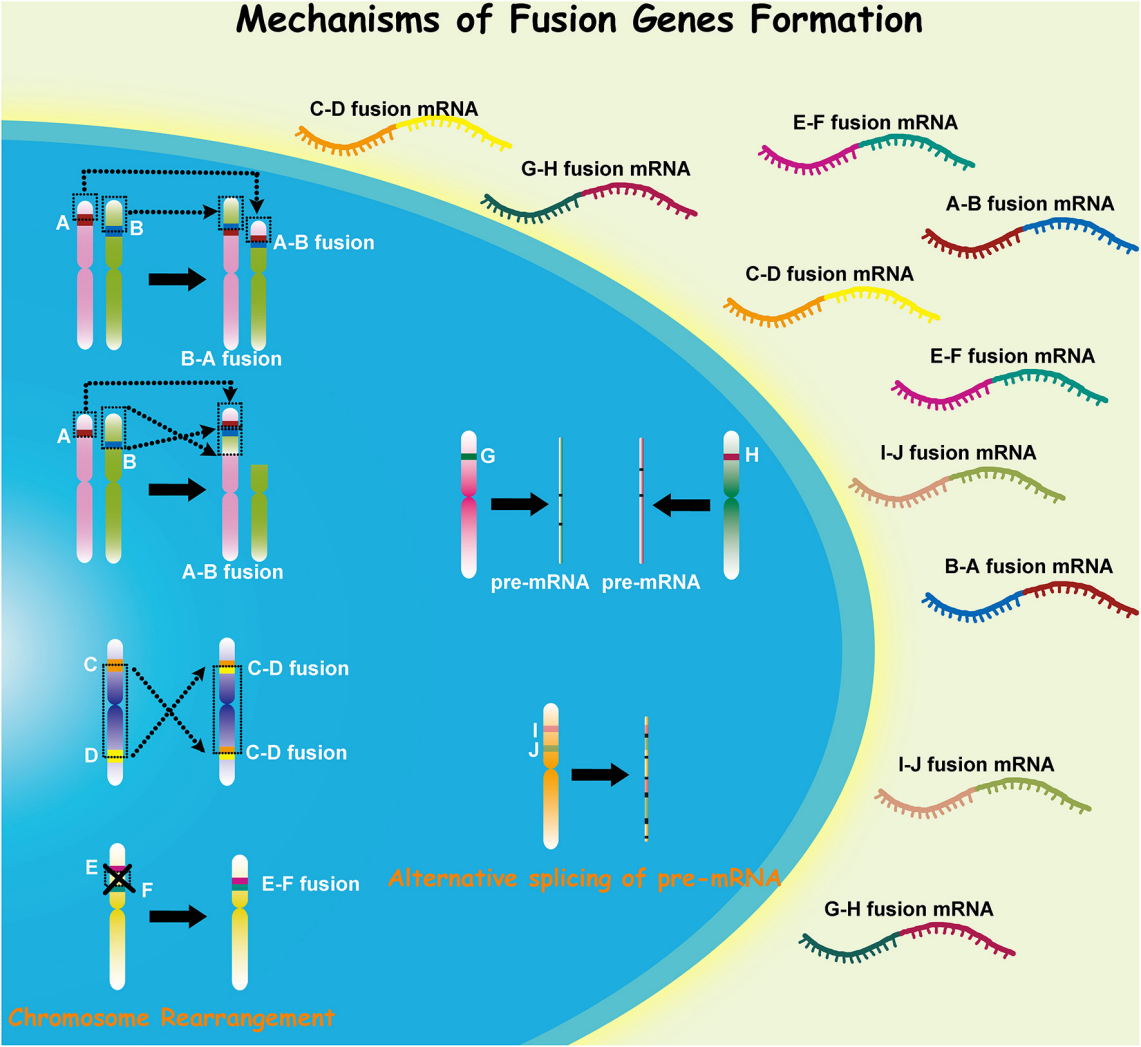
Different mechanisms in the formation of fusion genes.

Malignant glioma, which includes anaplastic gliomas and glioblastomas (GBMs), is the most common subtype of adult primary brain tumors. Despite recent therapeutic advances for this disease, it retains a notoriously deadly prognosis (1). Malignant gliomas are characterized by extensive genomic heterogeneity and instability (2, 3). With the increasing understanding of cancer pathogenesis and the development of high-throughput technologies in recent decades, the consequences of somatic mutations resulting in fusion genes have attracted more concerns (4, 5). Fusion genes have shown distinct capacity in both triggering and driving oncogenesis, and gene fusion can occur through various mechanisms in different cells (Figure 1). Investigation into the role of fusion genes in malignant gliomas has yielded promising yet still unrealized potential in the future management of these diseases.

The FIG-ROS1 fusion was the first identified gene fusion in GBM (6). Following its discovery, more fusion genes have been identified, many of which involve growth factor receptors. The EGFR-SEPT14 and EGFR-PSPH fusions have both been observed, with EGFR-SEPT14 being the most common fusion gene identified in GBM currently (7, 8). FGFR-TACC fusion is also commonly identified in anaplastic astrocytoma and GBM, and in mice this fusion has demonstrated explicit oncogenicity (9). Targeting fusion products has been a successful treatment strategy for other cancers, such as targeting BCR-ABL1 in chronic myeloid leukemia (10). Similarly, therapy targeted toward suppressing EGFR function has been shown to be an effective agent in treating GBM, and FGFR inhibition in mice harboring FGFR-TACC fusion has been shown to increase survival (8, 9). Notably, NTRK1 is a commonly found oncogene in various tumors while largely lacked in GBMs. However, NTRK1 fusions could play important roles in the oncogenesis in GBMs (11). Recently, PTPRZ1-MET fusion has been identified as an oncogenic mutation that can be observed in GBM and might provide useful new targets for future treatments (12).

The number of researches aimed at identifying and trying to understand the mechanisms of gene fusions in cancer is vast and will only continue to grow. The goal of this review is to identify all the reported fusion genes in malignant gliomas, collect and organize the relevant information mainly regarding gene fusions. The fusion genes that have been well characterized will be outlined as a way to guide future research on the less understood fusion mutations in malignant gliomas, of which more and more are being identified.

## Methods of Literature Search and Selection Criteria

A systematic review was performed following the Preferred Reporting Items for Systematic Review and Meta-Analyses of individual participant data (PRISMA-IPD) guidelines. PUBMED was searched for relevant studies published from their inception to March 16, 2021. The search strategy was a combination of the following MeSH terms: “glioma” or “astrocytoma” or “oligodendroglioma” or “oligoastrocytoma” or “glioblastoma” and “fusion”. The query results were managed by Endnote X9 software (Thomson Reuters, New York, NY, USA) and further screened, the exclusion criteria were listed as follows: 1) articles published in languages other than English; 2) meeting abstracts or abstract-only studies; 3) reviews, guidelines or classifications; 4) comments or letters; 5) case reports or small case series (<5 cases) unrelated to fusion genes; 6) studies unrelated to fusion genes; 7) studies in tumors other than adult supratentorial diffuse high-grade gliomas; 8) animal studies; 9) other irrelevant studies. Subsequently, potentially relevant literatures were obtained in full-text and assessed for eligibility, only studies focused on fusion genes in adult supratentorial diffuse high-grade gliomas would be included in final review.

## Results of Literature Search

**Figure 2:**
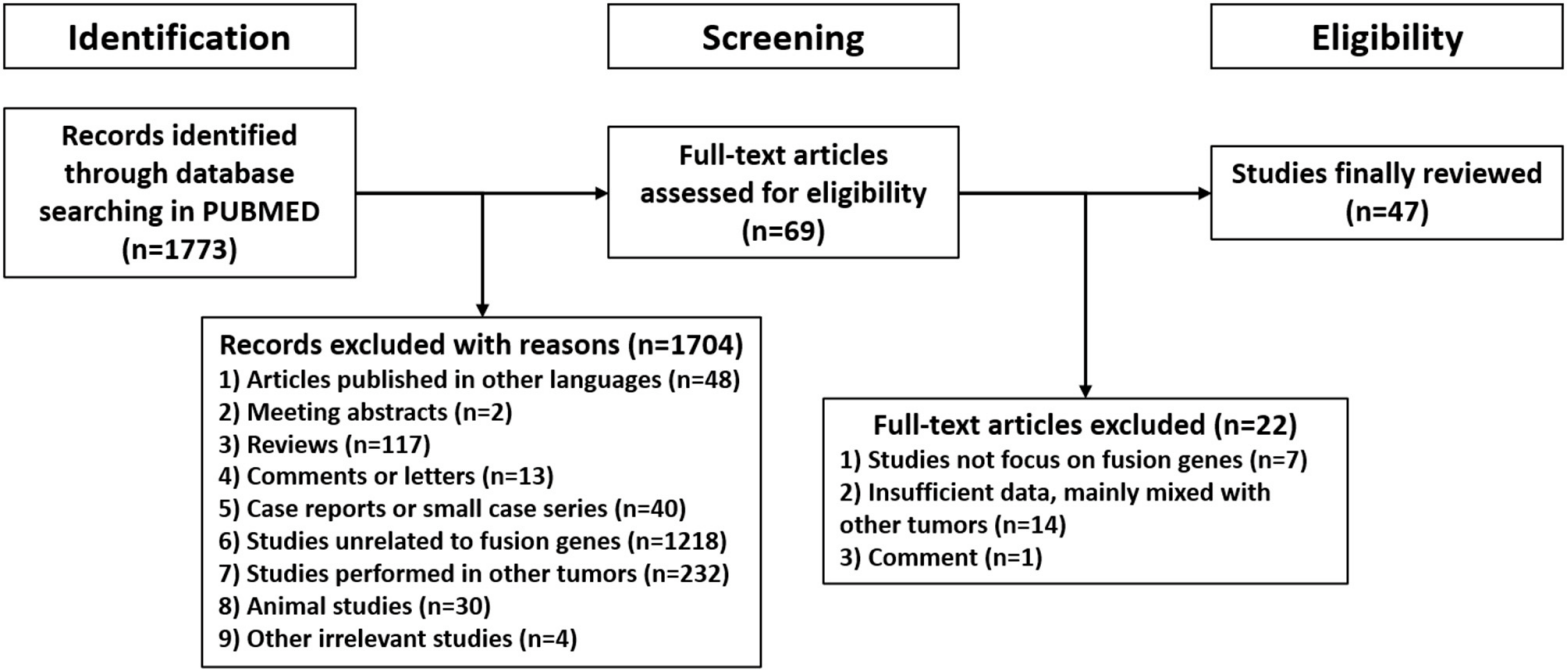
Flow chart of the systematic review.

**Figure 3:**
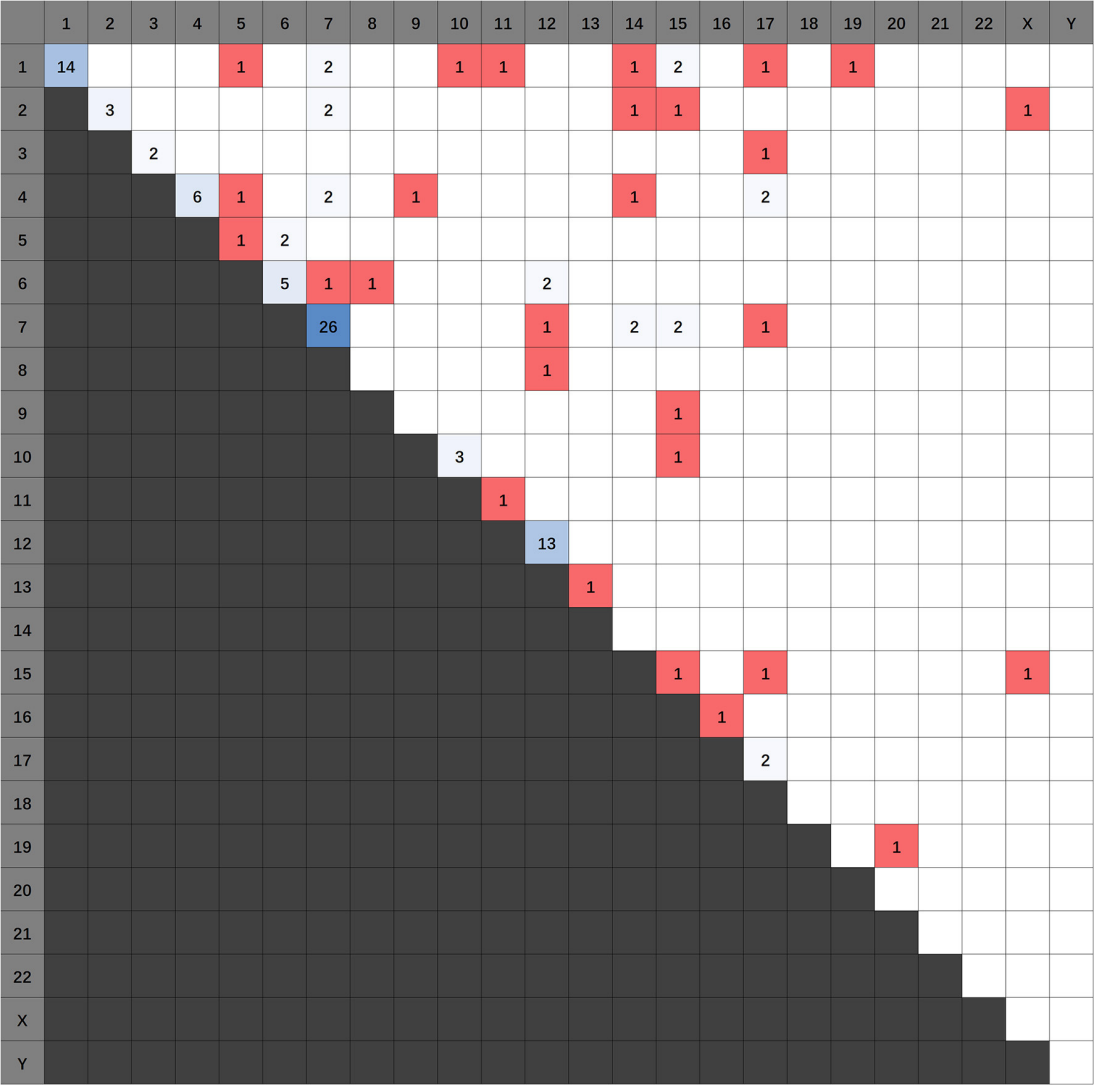
Heatmap detailing the chromosomal locations of the fusions identified.

1,773 publications were retrieved through a comprehensive literature search in PUBMED database. Subsequently, titles and abstracts of those articles were reviewed and screened. Ultimately, 69 articles were selected for full-text assessment, and 47 studies were finally reviewed. Figure 2 shows the flowchart. The selected studies comprised 120 identified fusion genes in adult supratentorial diffuse high-grade gliomas. Sixteen of them showed relative clear clinical significance, while the remaining 104 fusions were discovered just by screening analysis and there was little information regarding the oncologic implications of these proteins in the context of malignant gliomas. The lists for the two sets of fusion genes are summarized in Table 1 and Supplementary Table 1. An integrated heatmap presents the chromosomal relationships between those fusion partners (Figure 3), showing the top 3 most frequent chromosomal locations for gene fusions are chromosome 7, 1 and 12.

**Table 1:** Summary of fusion genes identified in adult supratentorial diffuse high-grade gliomas^*^.

**Fusion gene**	**Location**	**Significance**
BCAN-NTRK1	1q23.1-1q23.1	A potent oncogenic driver of high-grade gliomas, and is sensitive to TRK inhibitor entrectinib.
BCL6-RAF1	3q27.3-3p25.2	A novel RAF1-partnered fusion.
PSPHP1–ROS1	6q22.31-6q22.1	Potentially amenable for clinical intervention with kinase inhibitors.
EGFR-SEPT14	7p11.2-7p11.2	It can activate STAT3 signaling and confer mitogen independence and sensitivity to EGFR inhibition.
FGFR3-TACC3	4p16.3-4p16.3	It can generate an oncogenic protein that promotes tumorigenesis in GBM.
FIG-ROS1	6q22.1-6q22.1	It's frequency in gliomas remains controversial, but it plays oncogenic properties in many cancers.
GOPC–ROS1	6q22.1-6q22.1	Potentially amenable for clinical intervention with kinase inhibitors.
HMGA2-EGFR	12q14.3-7p11.2	It can activate EGFR signaling.
KANSL1-ARL17A	17q21.31-17q21.31	A cancer predisposition fusion gene associated with genetic backgrounds of European ancestry origin.
KDR-PDGFRA	4q12-4q12	It belongs to the immunoglobulin superfamily.
KIAA1549-BRAF	7q34-7q34	It is more frequent in oligodendroglial neoplasm and potentially responsible for deregulation of the Ras-RAF-ERK signaling pathway.
NFASC-NTRK1	1q32.1-1q23.1	It might have played driver role during the initiation or progression of the fusion-positive GBMs.
PTEN-COL17A1	10q23.31-10q25.1	It can regulate Collagen XVII expression.
PTPRZ1-MET	7q31.32-7q31.2	Highly enriched in secondary GBM, and has guiding significance for MET-targeted trials.
PTPRZ1-ETV1	7q31.32-7p21.2	A novel potential therapeutic target.

* *The clinical significance of these fusion genes is relatively clear*.

## Main Targetable Gene Fusions in Malignant Gliomas

### EGFR Fusions

#### Gene Location and Function

The epidermal growth factor receptor gene (EGFR) which lies on chromosome 7p11.2 is implicated in various cancers with some of the well-established examples including squamous cell carcinoma, epithelial cell line cancers, and GBM. The physiologic role of EGFR is to promote cellular growth, proliferation, and survival. Undoubtedly, alterations in such an anabolic pathway can result in oncogenesis.

#### Oncologic Implications of Altered EGFR Function

The estimated rate of EGFR amplification in GBM ranges from 25–40% (7, 13). In 20–30% of cases this is due to the variant EGFRvIII which results from a deletion of exons 2–7 leading to constitutive activity. The remainders of EGFR alterations are generally products of upstream gain of function mutations and fusions. Amplifications of EGFR are more common in tumors that have gene fusions in general, but the augmentation of EGFR can result from non-fusion events such as the case with EGFRvIII (8). Fusions of EGFR to either septin 14 (SEPT14) or phosphoserine phosphatase (PSPH) are present in 4–7% of GBM patients. Intriguingly, EGFR fusion events are less likely to co-occur with EGFRvIII, implying that a single one of the alterations is sufficient for oncogenesis (7).

The main mechanism by which EGFR fusions exert their mitogenicity is via activation of the STAT3 (signal transducers and activators of transcription 3) signaling pathway although in conjunction with other downstream products of EGFR activation that serve to promote growth and advancement of the cell cycle (14). The JAK (Janus Kinase)-STAT pathway is responsible for initiating transcription of regions of the genome that promote cellular growth and proliferation by inducing the expression of anti-apoptotic proteins and other cell cycle regulators (14). Additionally, activation of the EGFR pathway and specifically its localization to the nucleus bestows a degree of radioresistance and chemoresistance to the cell via induced expression of protective proteins (15, 16). In normal cells this pathway regulates the cell cycle, activating with growth is needed and deactivating when cell cycle arrest is appropriate (17). STAT3 in particular has been implicated in a number of other cancers including those arising from epithelial cell lines such breast cancer, lung cancer, bladder cancer as well as hematologic cancers and aberrations such as acute myeloid leukemia, diffuse large B-cell lymphoma, large granular lymphocytic leukemia, aplastic anemia, and myelodysplastic syndrome (18). For hematologic cancers in particular, targeting the overactivity of STAT3 with inhibitors has shown promise (19). The most common EGFR fusion in GBM, EGFR-SEPT14 is detailed below.

#### EGFR-SEPT14 Fusion

EGFR-SEPT14 fusion was the most observed gene fusion in GBM (4%). However, preliminary evidence suggests that it can be also present in Grade II and Grade III gliomas possessing wild-type isocitrate dehydrogenase (IDH). The fusion occurs following an in-frame fusion event with loss of the C-terminal domain of EGFR to the 9th intron of SEPT14 which also lies on chromosome 7 (7, 8). SEPT14 is a testis specific member of the GTP-binding proteins of the cytoskeleton known collectively as septins, and it is implicated in membrane transport, apoptosis, cell polarity, cell cycle regulation, and cytokinesis (20). The result is fusion protein that possesses the tyrosine kinase domain of EGFR and the coiled-coil domain of SEPT14. Furthermore, as Frattini et al. demonstrated, this fusion is sufficient to confer mitogen independence to the cell that possess this fusion which if unchecked progresses to carcinoma. Nevertheless, at this time, it is unknown how EGFR-SEPT14 will play into diagnosing, prognosticating, and treating patients (21).

#### EGFR-PSPH Fusion

The second most frequent fusion of EGFR is the EGFR-PSPH fusion (2.2%). Analysis of The Cancer Genome Atlas (TCGA) has indicated that the same N-terminal portion of EGFR is implicated; however, in place of SEPT14, PSPH serves as the 3' partner (7). PSPH, which lies on chromosome 7p11.2, encodes the protein phosphoserine phosphatase which is responsible for the hydrolysis of L-phosphoserine as well as involved in additional aspects of amino acid metabolism (22). Similar to EGFR-SEPT14, the fusion partner of EGFR merely serves as a vessel in order to increase the expression of EGFR. The clinical role and characterization of EGFR-PSPH have not yet to be extensively explored.

#### HMGA2-EGFR Fusion

A novel fusion gene HMGA2-EGFR has been identified by Komuro et al. (23). It could encode a protein comprising the N-terminal region of the high-mobility group AT-hook protein 2 (HMGA2, chromosome location 12q14.3) fused to the C-terminal region of EGFR. The fusion products represented transforming potential and high tumor-forming capacity in cell culture. Compared with EGFRvIII, HMGA2-EGFR could induce a higher level of phosphorylated STAT5B. Further investigation remains required to explore the clinical role and characterization of this novel fusion gene.

#### Targeting EGFR Fusions and Directions for the Future

Tyrosine Kinase Inhibitors (TKIs) like lapatinib and erlotinib are the most clinically popular EGFR-targeting therapy to date (24). A previous *in vitro* study showed that the mitogenic capacity granted by EGFR-SEPT14 is reversible via TKIs, providing some exciting insight into treating GBMs with EGFR-SEPT14 fusions (7). But overall, there is still a long way before targeting EGFR fusions can be applied to clinical practice. In addition, clinical trials did not show significant survival benefits of EGFR inhibitors in GBM patients, and the non-specific patient selection might be the underlying main reason. Future studies may focus on looking for effective therapeutic targets and selecting patients through individual genomic examination.

### FGFR-TACC Fusions

#### Gene Function of FGFR

The fibroblast growth factor receptor (FGFR) family that interacts with fibroblast growth factor (FGF) ligands is a highly evolutionarily conserved mediator of a variety of physiologic functions. Some of the functions of the FGFR family include signaling cellular stages of development, preserving homeostasis, as well as policing metabolic processes (25). Mutations of the FGFR family of receptors have been implicated in a number of different cancers including lung, cervical and bladder cancers in addition to GBM (26). The four main human FGFR receptor types are numerically named FGFR1, 2, 3, and 4. FGFR1, FGFR2, and FGFR3 are the most germane to the discussion of glioma associated fusion genes with the majority of fusion events involving FGFR1 (9, 27, 28).

#### Oncologic Implications of FGFR-TACC Fusions

FGFR-TACC is the best described of the FGFR fusions in gliomas. The rate of FGFR2/3-TACC fusion in GBM is estimated to be on the order of 2.6-10% with most estimates in the 3% range (9, 29, 30). Abstractly, this fusion occurs with an FGFR locus and its corresponding TACC located on the same chromosome. The result is a constitutively phosphorylated fusion product that exerts oncogenic effect (9). For instance, the most commonly observed fusion is FGFR3-TACC3 both located on chromosome 4. Yet, fusions of FGFR2 and TACC2 along with FGFR1 and TACC1 located on chromosomes 10 and 8 respectively have been reported as well. This fusion event is possible owing to the proximity of FGFR and TACC on their respective chromosomes (9). The TACC protein is also a highly evolutionarily conserved protein with a distinctive coiled-coil domain similar to that of SEPT14 involved in EGFR fusions. Its overexpression is present in malignancies such lung cancer, colon cancer, and multiple myeloma (31). TACC's physiologic function is to localize to and stabilize the mitotic spindle during mitosis, specifically during chromosomal separation (32, 33). When fused, these two proteins can wreak havoc on the individual genome of a cell culminating in tumor formation in a manner independent of FGF ligands.

Singh et al. demonstrated a potential mechanism by which FGFR fusions exert their oncogenic effect. Their analysis was specifically focused on the FGFR-TACC fusion; however, it has been postulated that other FGFR fusions (e.g. FGFR3-ELAVL) exert their effect by a similar mechanism (3). They performed their experiments both in the presence of FGF ligands and without and noted that FGF presence was not necessary for effect but in fact due to constitutive phosphorylation of the tyrosine kinase (TK) domain of the fusion complex acting in a manner distinct from the physiologic pathway of FGFR. Experiments with inactivated TK domains did not demonstrate oncogenic capacity suggesting that this is the underlying mechanism for the effect of FGFR fusions (9). The result of this effect was aberrant chromosome segregation during mitosis leading to general chromosomal instability and aneuploidy (9, 27). Once destabilized, the chromosomes lose genetic fidelity during replication ultimately resulting in oncogenesis (27). Frattini et al. elucidated that FGFR3-TACC3 fusions could activate oxidative phosphorylation and mitochondrial biogenesis and induce sensitivity to inhibitors of oxidative metabolism. It uncovers an oncogenic circuit engaged by FGFR-TACC fusions in cancer (34).

#### Targeting FGFR-TACC Fusions and Directions for the Future

As far as the clinical role of FGFR-TACC fusions, there are a number of small molecule and antibody inhibitors that target the FGFR axis. The oldest class of drugs that target this axis are the Oxindoles, discovered in 1997. Other drug classes that target FGFR and have been examined for their potential anti-cancer properties include the Pyrido (2,3-d) pyrimidines, Quinolines, Azaindoles, Indazoles, Naphthyridines, Pyrrolo (2,1-f) (1, 2, 4) triazines, and a variety of antibodies targeting different sites of the FGFR receptor. However, FGFR inhibitors were not developed specifically for brain tumors. The existence of blood-brain barrier remains a challenge for FGFR-TACC targeting and only recently has FGFR become a focus for targeted glioma therapy.

Drugs like dovitinib are currently in Phase I trials for patients with recurrent GBM. This drug takes the wide approach and targets FGFR, VEGFR, PDGFR β, and c-Kit. Unfortunately, in the Phase I cohort the FGFR-TACC fusion was unable to be detected in the group of patients with progression free survival after 6 months. However, it is unclear whether this observed effect was a product of the interaction between the drug and tumor genetics or merely a result of the small size of the Phase I patient cohort (35). BGJ398 is an FGFR inhibitor originally studied for epithelial cancers that has recently entered Phase II trials (NCT01975701) for GBM patients with FGFR amplifications and fusions (36). Erdafitinib (JNJ-42756493) is a small molecule pan-FGFR inhibitor that shows promise for specifically inhibiting FGFR3-TACC3 fusions in IDH wild-type glioma *in vitro* as well as in a Phase I clinical trial (NCT01703481) (37). It is thought that erdafitinib exerts its effect by inducing selective radiosensitivity of tumor cells (27). Although the high heterogenicity and the indefinable longitudinal evolutionary path of GBM are still obstacles for successful FGFR-TACC targeting, cautious optimism should be maintained for future studies (38).

### NTRK Fusions

NTRK encodes the tropomyosin receptor kinase (TRK) receptor family. They actively participate in neuronal development, maintenance, and protection. The rearrangements of TRK receptor family play important parts in the oncogenesis in various cancers including glioma. These fusion proteins may induce tumor cell proliferation and activate downstream PI3K-AKT, RAS/MAPK/ERK signaling pathways. Given the little understanding in the function of NTRK2/3 fusions in glioma, we emphatically describe NTRK1 fusions here.

#### NTRK1 Gene Partner Locations and Functions

NTRK1 is a known oncogene located in 1q23.1. It encodes a kinase member of the NTRK family, which is also a high affinity receptor for nerve growth factor (NGF). RNA-Seq data of TCGA showed that NTRK1 was observed to be fused with two genes, neurofascin (NFASC, location 1q32.1) and brevican (BCAN, location 1q23.1) (11). Another two novel in-frame fusions of NTRK1, CHTOP-NTRK1 and ARHGEF2-NTRK1, were found by Zheng et al. (39), and the biological functions of the two novel fusion partners are not clear yet.

#### Oncologic Implications of NTRK1 Fusions

NTRK1 gene fusions indicate not only elevated expression of NTRK1, but also NGF-triggered activation of the NGF/TrkA downstream pathway. The involvement of NTRK1 in GBM remains unknown, however, it is frequently involved in other cancers. Most of the genes partner of NTRK1 harbor coiled-coil domains, which could mediate dimerization of the fusion genes and consequent activation of the TrkA kinase domain. NFASC and BCAN are two more exceptions without coiled-coil domains. Ig-like domains appear to mediate dimerization of TrkA instead of coiled-coil domains. TrkA has Ig-like domains within the extracellular portion of the protein, which mediate NGF-dependent dimerization. In addition, transduction of the NFASC-NTRK1 fusion gene can result in increased proliferation in cell model (11).

#### Targeting NTRK Fusions and Directions for the Future

TRK inhibitors have already shown potential efficacy in tumors with functional NTRK fusions, including gliomas. Entrectinib (RXDX-101) is a pan-TRK inhibitor. A preclinical study showed the efficacy of entrectinib on GBM in a mouse model with BCAN-NTRK1 fusions (40). In a Phase II basket study (STARTRK-2), the therapeutic effects of entrectinib were evaluated in patients suffering from different tumors, and the only glioma patient with BCAN-NTRK1 fusion represented almost a halving in tumor volume (41). Larotrectinib (LOXO-101) is a selective pan-TRK inhibitor. Preliminary results from NAVIGATE Phase 2 larotrectinib trial (NCT02576431) showed its significant role in treating NTRK fusion-positive recurrent GBM (42). Larotrectinib was also considered to have marked and durable antitumor activity in patients with TRK fusion-positive cancer (NCT02576431 and NCT02637687) (43). Although the biological mechanism and clinical significance of NTRK fusions remain unclarified, further studies and clinical trials are needed for targeted therapy in GBM patients regarding NTRK fusions.

### MET Fusions

#### Gene Partner Locations and Functions

MET is a well characterized transmembrane receptor tyrosine kinase implicated in a number of cancers from non-small-cell lung cancers and solid organ tumors like papillary renal cell carcinoma and hepatocellular carcinoma to various head and neck cancers in addition to CNS tumors. MET's expression is normally tightly delimited by numerous mechanisms including epigenetic modifications, DNA methylation, transcriptional regulators, post-transcriptional glycosylation and phosphorylation, as well as interaction with growth factors such as the Hepatocyte Growth Factor (HGF) ligand (44).

The PTPRZ1-MET (ZM) fusion is a recently discovered gene fusion of GBM. This fusion occurs as a result of intron insertion and tandem duplication between the protein tyrosine phosphatase receptor type Z1 (PTPRZ1) gene located on chromosome 7q31.32 and the closely located MET proto-oncogene receptor tyrosine kinase (MET, location 7q31.2). This insertion can result in both in-frame and out of frame transcripts depending on the location of gene insertion during fusion (12). PTPRZ1 is only expressed in the CNS and is thought to be responsible for CNS development and repair following injury. Its expression is commonly altered in a variety of cancers including GBM and other non-CNS tumors (45). And while its behavior is characterized, its utility as a clinical tool is yet to be fully characterized. PTPRZ1-MET bypasses many of these regulatory mechanisms resulting in overexpression of MET and subsequent activation of the MET signaling pathway.

#### Oncogenic Implications of MET Overactivity

Overexpression of MET results in a wide variety of downstream effects culminating in oncogenesis. Physiologically, MET is responsible for cellular growth and proliferation in response to HGF, so it is no surprise that MET overexpression can result in tumorigenesis. The overexpression of MET exerts its oncogenic capacity in two main ways: by providing additional binding sites for HGF and undergoing ligand independent dimerization and activation (46). Once activated, MET effects a number of known oncogenic pathways namely, RAS, PI3K, and JAK-STAT. Dysregulation of these pathways results in tumor growth via self-sustaining neurosphere formation, angiogenesis, and after sufficient time ability for tumor to metastasize (45, 47–49). PTPRZ1-MET exerts its oncogenetic capacity by hijacking the MET pathway resulting in tumor formation and if left unchecked, metastasis.

#### Targeting MET Fusions and Directions for the Future

Hu et al. demonstrated that MET-exon-14-skipping frequently co-occurred with ZM fusions and was present in about 14% of secondary GBM patients with significantly worse prognosis. As a MET kinase inhibitor, PLB-1001 had remarkable potency in selectively inhibiting MET-altered tumor cells in preclinical models and clinically achieved partial response in some advanced secondary GBM patients (NCT02978261) (50). In another study by Bender et al. they treated a pediatric patient bearing a MET-fusion-expressing GBM with the targeted inhibitor crizotinib. The therapy led to substantial tumor shrinkage and associated relief of symptoms (51). These clinical findings indicate a clinical potential for precisely treating gliomas by targeting MET fusions.

### FIG-ROS1 Fusion

#### Gene Partner Locations and Functions

The human ROS1 gene (location 6q22.1) was initially discovered as the homolog of the chicken c-ros. ROS1 encodes for a receptor tyrosine kinase (RTK), which is most closely related to the ALK and LTK human RTKs (52), is recently shown to be involved in genetic rearrangements with transforming capability. Endogenous ROS1 rearrangements were first observed in the human GBM cell line U118MG, in which an interstitial deletion of 240 kilobases on 6q21 region is responsible for the fusion of exon 7 of FIG (Fused In GBM, location 6q22.1) (also known as GOPC, Golgi-associated PDZ and coiled-coil motif containing) with exon 35 of ROS1. FIG encodes for a 454-amino acid protein that includes a PSD-95, Disc Large, ZO-1 (PDZ) domain, two coiled-coil regions, and a leucine zipper. FIG associates peripherally with the Golgi apparatus by interacting through its second coiled-coil domain with a SNARE protein and may play a role in oncogenic signaling (6).

#### Oncologic Implications of FIG-ROS1 Fusion

The FIG-ROS1 transcript is encoded by 7 FIG exons and 9 ROS-derived exons. In mice, the FIG-ROS1 fusion gene has been shown to promote the formation of astrocytomas when ectopically expressed in the basal ganglia (53). The FIG-ROS1 locus encodes for an in-frame fusion protein with a constitutively active kinase activity. Expression of the FIG-ROS1 fusions in GBM or fibroblasts cells has been shown to result in auto-phosphorylation of ROS1 and phosphorylation of SHP-2, MEK, ERK, STAT3, and AKT (53, 54).

#### Targeting FIG-ROS1 Fusion and Directions for the Future

Despite the delineation of the FIG-ROS1 rearrangements is rare in clinical cases with glioma (55, 56), the prospects of targeting FIG-ROS1 fusions should not be neglected. Experimental use of combination therapy consisting of crizotinib and temozolomide to desensitize and target FIG-ROS1 fusions in cell cultures from adult GBM has had a profound antitumor effect *in vitro* and *ex vivo* (57). Davare et al. demonstrated that lorlatinib, an ROS1 inhibitor, significantly prolonged survival in an intracranially xenografted tumor model generated from a ROS1 fusion-positive GBM cell line (58). Crizotinib is an FDA-approved ROS1 inhibitor that could potentially target the FIG-ROS1 fusion and is being used as salvage therapy for cancers. Ensartinib, as another targeted medicine to ROS1 fusion, is currently enrolled into a phase II Pediatric MATCH trial containing refractory CNS Neoplasm (NCT03213652). Further development of treatment guidelines for ROS1 inhibitors may represent a promising modality for future study.

### Other Fusions in GBMs

Ozawa et al. investigated the PDGFRA locus in PDGFRA-amplified gliomas and identified the first case of a gene fusion between kinase insert domain receptor (KDR) (VEGFRII) and the PDGFRA gene (KP fusion) (59). Tumors with this fusion displayed histologic features of oligodendroglioma. The authors subsequently demonstrated the fusion proteins was autophosphorylated on tyrosine residues and associated with the activation of downstream MAPK and PI3K signaling pathways. These results suggest the possibility that KP fusion behaves as oncogene in PDGFRA-amplified GBMs.

Shah et al. explored genomic data of 185 GBM samples and identified 27 fusion gene partners, including some novel non-coding genes, such as non-coding RNA RP11-745C15.2 fused with LANCL2 gene (8). Moreover, RNA RP11-745C15.2 was also found to be fused with EGFR. Both fusions can lead to C-terminal truncation of the fused gene. The underlying signaling pathway needs to be further investigated.

Subramaniam et al. reported a total fusion incidence of 9.7% in 404 glioma tumor specimens tested by RNASeq analysis. Some of them haven't been previously described in gliomas (e.g., EGFR-VWC2, FGFR-NBR1, FGFR-BRAP, ST7-MET, RAB3IP-PDGFRA and several NTRK2 fusions) (60). Additionally, fusion genes, such as MAN2A1–FER, CCNH-C5orf30, TRMT11-GRIK2, were discovered in multiple cancer types including GBMs (61, 62). The underlying function of these fusions in gliomas needs to be further investigated. To guide novel strategies of targeted therapy, more experimental and clinical trials are essential for further understanding these newfound fusion genes.

## Conclusions

**Figure 4:**
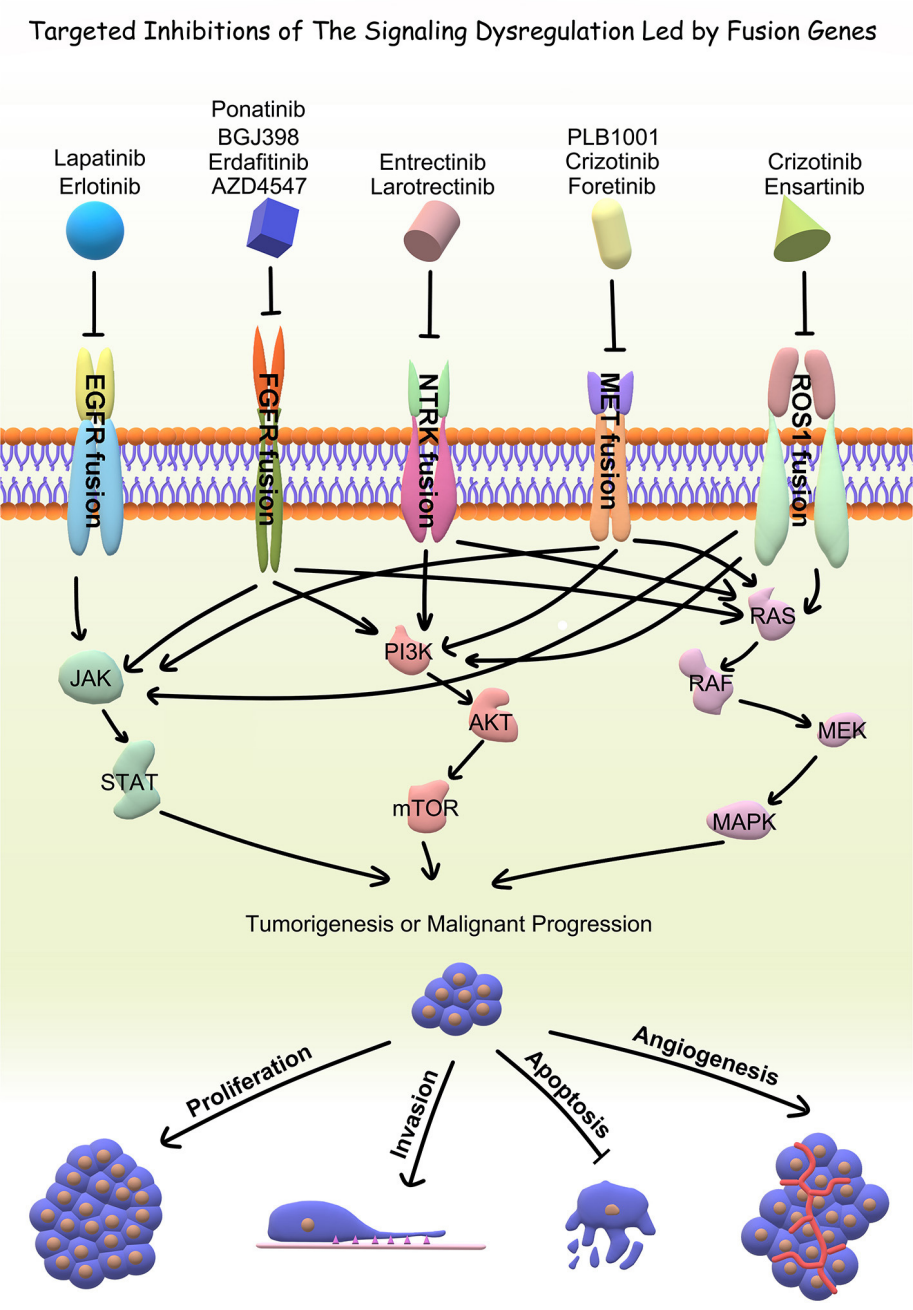
Downstream pathways related to specific driver fusions and targeted inhibitors.

With the advent of rapid DNA and RNA sequencing, proteomics and bioinformatics, recent literature has done a wonderful job in identifying and characterizing fusion partners and transcripts. However, the effects of fusion genes on tumor biological behavior and relevant internal mechanism are far from clarified, which limits further exploration on the diagnostic and therapeutic value of fusion genes. Accordingly, the functional characterization of fusion genes should be the next step for translating the existing wealth of information to the clinical setting. In the current review, we summarized fusion genes with relatively clear biological characteristics in adult malignant gliomas (Table 2). Identification of such fusion genes and associated kinases may allow us to exploit therapeutic opportunities with targeted therapies in adult malignant gliomas. Targeted drugs have great promise to be applied directly to malignant gliomas subject to the oncogenic fusions (Figure 4). Relative clinical trials are still ongoing in recurrent high-grade gliomas (Table 3) and some of them presented exciting preliminary findings. In the coming era of integrated diagnosis and personalized treatment for gliomas, to identify more fusion genes as biomarkers across different glioma subtypes and to apply corresponding targeted therapy is an inexorable trend.

**Table 2:** Biological characteristics of recurrent gene fusions as therapeutic targets in GBM.

**Fusion** **gene**	**Fusion** **incidence**	**Chromosome** **location**	**Main gene** **partners**	**Signaling** **pathway**	**Targeted** **medicine**
EGFR	2.2–4%	7p11.2	SEPT14 PSPH HMGA2 VWC2	STAT3STAT	Lapatinib, Erlotinib
FGFR1FGRR3	1.1% 1.2–8.3%	8p11.234p16.3	TACC1 TACC 3 BRAP NBR1	ERK, MAPK, PI3K, and JAK-STAT	Ponatinib BGJ398, Erdafitinib, AZD4547
NTRK1NTRK2NTRK3	1.2–1.7%	1q23.1	NFASC BCAN CHTOP ARHGEF2 GKAP KCTD8 NOS1AP SQSTM1 TBC1D2 VCAN EML4	NGF/TrkA	Entrectinib, Larotrectinib
MET	3%	7q31.2	PTPRZ1 TGF CLIP2 CAPZA2 ST7 TPR	HGF, MAPK, PI3K, STAT	PLB1001, Crizotinib, Foretinib
ROS1	<0.6%	6q22.1	FIG(GOPC)	SHP-2, MAPK, PI3K, STAT	Crizotinib, Ensartinib

**Table 3:** Ongoing clinical trials involving fusion genes in GBM.

**Fusion gene**	**Targeted medicine**	**NTC number**	**Phase**	**Number** **enrolled**	**Status**	**Study title**
FGFR fusion	Anlotinib	NCT04004975	I/II	50	Recruiting	Clinical study on the treatment of recurrent Glioblastoma with anlotinib
	BGJ398	NCT01975701	II	26	Completed	A phase 2 study of BGJ398 in patients with GBM
	Erdafitinib (JNJ-42756493)	NCT01703481	I	188	Completed	A study to evaluate the safety, pharmacokinetics, and pharmacodynamics of JNJ-42756493 in adult participants with advanced or refractory solid tumors or lymphoma
	AZD4547	NCT02824133	I/II	14	Completed	Treatment with AZD4547 for recurrent malignant glioma expressing FGFR-TACC gene fusion
NTRK fusion	Larotrectinib (LOXO-101)	NCT02576431	II	320	Recruiting	A study to test the effect of the drug Larotrectinib in adults and children with NTRK-fusion positive solid tumors (NAVIGATE)
		NCT02637687	I/II	104	Recruiting	A study to test the safety and efficacy of the drug Larotrectinib for the treatment of tumors with NTRK-fusion in children (SCOUT)
		NCT03213704	II	49	Recruiting	Larotrectinib in treating patients with relapsed or refractory advanced solid tumors, non-hodgkin lymphoma, or histiocytic disorders with NTRK Fusions (a pediatric MATCH treatment trial)
		NCT03834961	II	70	Not yet recruiting	Larotrectinib in treating patients with previously untreated TRK fusion solid tumors and TRK fusion relapsed acute leukemia
NTRK fusion/ROS1 fusion	Entrectinib (RXDX-101)	NCT02650401	I/II	65	Recruiting	Study of entrectinib (Rxdx-101) in children and adolescents with no curative first-line treatment option, recurrent or refractory solid tumors and primary cns tumors, with or without Trk, Ros1, or Alk fusions
		NCT02568267	II	300	Recruiting	Basket study of Entrectinib (RXDX-101) for the treatment of patients with solid tumors harboring NTRK 1/2/3 (Trk A/B/C), ROS1, or ALK gene rearrangements (Fusions) (STARTRK-2)
PTPRZ1-MET fusion	PLB1001	NCT02978261	I	20	Recruiting	Study of a c-Met inhibitor PLB1001 in patients with PTPRZ1-MET fusion gene positive recurrent high-grade glioma
MET fusion/ ROS1 fusion	Crizotinib	NCT02270034	I	24	Active, not recruiting	Study to evaluate safety and activity of crizotinib with temozolomide and radiotherapy in newly diagnosed glioblastoma
ROS1 fusion	Ensartinib	NCT03213652	II	98	Recruiting	Ensartinib in treating patients with relapsed or refractory advanced solid tumors, non-hodgkin lymphoma, or histiocytic disorders with ALK or ROS1 genomic alterations (a pediatric MATCH treatment trial)

## Author Contributions

GY conceptualized the review. GY, XF, and HH performed the systematic review and wrote the manuscript. GY and XF prepared the figures and tables. TJ and CC critically reviewed the manuscript. All authors read and approved the final manuscript.

## Funding

This work was supported by the National Natural Science Foundation of China (No. 81871013), Beijing Municipal Education Commission Science and Technology Plan General Project (No. 1192050172), and Beijing Tiantan Hospital Young Scientist Program (YSP201705).

## Conflict of Interest

The authors declare that the research was conducted in the absence of any commercial or financial relationships that could be construed as a potential conflict of interest.

## Publisher's Note

All claims expressed in this article are solely those of the authors and do not necessarily represent those of their affiliated organizations, or those of the publisher, the editors and the reviewers. Any product that may be evaluated in this article, or claim that may be made by its manufacturer, is not guaranteed or endorsed by the publisher.

## Abbreviations

GFR: Epidermal growth factor receptor
FGFR: Fibroblast growth factor receptor
FIG: Fused In GBM
GBM: Glioblastoma multiforme
HGF: Hepatocyte Growth Factor
HMGA2: High-mobility group AT-hook protein 2
IDH: Isocitrate Dehydrogenase
JAK: Janus Kinase
PSPH: Phosphoserine phosphatase
PTPRZ1: Protein tyrosine phosphatase receptor type Z1
RTK: Receptor Tyrosine Kinase
SEPT14: Septin 14
STAT3: Signal transducers and activators of transcription 3
TK: Tyrosine Kinase
TKI: Tyrosine Kinase Inhibitors
TRK: tropomyosin receptor kinase.
