# Supplementary material for: Fusion Genes Altered in Adult Malignant Gliomas
Source: Front Neurol. 2021 Oct 4;12:715206. doi: 10.3389/fneur.2021.715206 (PMC8520976; doi:10.3389/fneur.2021.715206)
Supplement: Supplementary file 1 [file Table_1.DOC]

**Table S1** Summary of fusion genes identified in adult supratentorial diffuse high-grade gliomas^*^

| **Fusion Gene** | **Location** |
| --- | --- |
| ABCD3-ANUBL1 | 1p21.3-10q11.22 |
| ABCD3-INTS2 | 1p21.3-17q23.2 |
| ACSL6-DNAH8 | 5q31.1-6p21.2 |
| ADAMTS17-LPAR1 | 15q26.3-9q31.3 |
| AGK-BRAF | 7q34-7q34 |
| ANK2-SLC26A5 | 4q-7q22.1 |
| ANK2-TRIM9 | 4q-14q22.1 |
| ANKS1A-SLC26A5 | 6p21.31-7q22.1 |
| ARHGEF2-NTRK1 | 1q22-1q23.1 |
| ARID1A-BEND5 | 1p36.11-1p33 |
| ARID1A-RNF31 | 1p36.11-14q12 |
| ARID4B-GNG4 | 1q42.3-1q42.3 |
| ASH1L-C1orf61 | 1q22-1q22 |
| BLVRA-SEPT14 | 7p13-7p11.2 |
| C12orf49-MDM2 | 12q24.22-12q15 |
| CAPZA2-MET | 7q31.2-7q31.2 |
| CCDC6-RET | 10q21.2-10q11.21 |
| CCNH-C5orf30 | 5q14.3-5q21.1 |
| CEP85L-SYTL3 | 6q22.31-6q25.3 |
| CHTOP-NTRK1 | 1q21.3-1q23.1 |
| CPA6-CPM | 8q13.2-12q15 |
| CPM-MDM2 | 12q15-12q15 |
| CREB1-PARD3B | 2q33.3-2q33.3 |
| CRTC1-ABHD12 | 19p13.11-20p11.21 |
| CSDE1-CDC73 | 1p13.2-1q31.2 |
| CTDSP2-LINC02532 | 12q14.1-6q21 |
| EGFR-PPM1H | 7p11.2-7p11.2 |
| EGFR-PSPHP1 | 7p11.2-7p12.2 |
| EGFR-VOPP1 | 7p11.2-7p11.2 |
| EGFR-VSTM2A | 7p11.2-7p11.2 |
| EGFR-VWC2 | 7p11.2-7p12.2 |
| EIF4H-GTF2I | 7q11.23-7q11.23 |
| EML4-NTRK3 | 2p21-15q25.3 |
| FAM111B-FAM111A | 11q12.1-11q12.1 |
| FGFR3-BRAP | 4p16.3-4p16.3 |
| FGFR3-CAMK2A | 4p16.3-5q32 |
| FGFR3-NBR1 | 4p16.3-17q21.31 |
| FRS2-DTX3 | 12q15-12q13.3 |
| FRS2-KIF5A | 12q15-12q13.3 |
| GIGYF2-ECEL1 | 2q37.1-2q37.1 |
| GIGYF2-PPP1R7 | 2q37.1-2q37.3 |
| HMGA2-NUP107 | 12q14.3-12q15 |
| INTS2-MED13 | 17q23.2-17q23.2 |
| KLHL7-BRAF | 7p15.3-7q34 |
| LANCL2-PSPH | 7p11.2-7p11.2 |
| LANCL2-SEPT14 | 7p11.2-7p11.2 |
| LANCL2-VSTM2A-OT1 | 7p11.2-7p11.2 |
| LEMD3-CPM | 12q14.3-12q15 |
| LEO1-SLC12A1 | 15q21.2-15q21.1 |
| LRP12-PARK2 | 8q22.3-6q26 |
| MON2-MARS1 | 12q14.1-12q13.3 |
| MTOR-TP53BP1 | 1p36.22-15q15.3 |
| MYBPHL-FAM63B | 1p13.3-15q |
| NAA15-LPAR1 | 4q31.1-9q31.3 |
| NAB2-STAT6 | 12q13.3-12q13.3 |
| NF1-SH3GL3 | 17q11.2-15q25.2 |
| NFASC-PRELP | 1q32.1-1q32.1 |
| NFASC-RTN3 | 1q32.1-11q13.1 |
| NFASC-SOX13 | 1q32.1-1q32.1 |
| NUDT9-EFTUD2 | 4q22.1-17q21.31 |
| PANK4-DCST1 | 1p36.32-1q21.3 |
| PIK3C2B-DSTYK | 1q32.1-1q32.1 |
| PLEKHA6-PIK3C2B | 1q32.1-1q32.1 |
| PPIL3-PTPN12 | 2q33.1-7q11.23 |
| PPM1H-MDM2 | 12q14-12q15 |
| PPP1R9A-PSMC2 | 7q21.3-7q22.1 |
| PPP2R2B-CCT3 | 5q32-1q22 |
| PTPN12-LUC7L2 | 7q11.23-7q34 |
| RAB7A-FOXP1 | 3q21.3-3p13 |
| RARB-EFTUD2 | 3p24.2-17q21.31 |
| SCFD2-CLOCK | 4q12-4q12 |
| SEC61G-EGFR | 7p11.2-7p11.2 |
| SERINC5-SCUBE3 | 5q14.1-6p21.31 |
| SH3RF3-HUWE1 | 2q13-Xp11.22 |
| SH3RF3-TRIM9 | 2q13-14q22.1 |
| SLC26A5-ZNF839 | 7q22.1-14q32.31 |
| SND1-TMEM178B | 7q32.1-7q34 |
| ST7-MET | 7q31.2-7q31.2 |
| TBC1D14-HTRA3 | 4p16.1-4p16.1 |
| TDRD3-ESD | 13q21.2-13q14.2 |
| TLL2-FAM63B | 10q24.1-15q |
| TMEM91-TAL1 | 19q13.2-1p33 |
| TRMT11-GRIK2 | 6q22.32-6q16.3 |
| TSFM-IFNG | 12q14.1-12q15 |
| TSFM-TJAP1 | 12q14.1-6p21.1 |
| VOPP1-ABCA13 | 7p11.2-7p12.3 |
| VOPP1-SEPT14 | 7p11.2-7p11.2 |
| VTI1A-TCF7L2 | 10q25.2-10q25 |
| WDR90-RHOT2 | 16p13.3-16p13.3 |
| XRCC6BP1-SRRM4 | 12q14.1-12q24.23 |
| YEATS4-SLC35E3 | 12q15-12q15 |
| YEATS4-XRCC6BP1 | 12q15-12q14.1 |
| (NM_001130143)-PCDH11X | 15-X |
| (NM_001131008)-LINGO1 | 7-15 |
| (NM_001131009)-MED13 | 7-17 |
| (NM_001131009)-RSBN1L | 7-7 |
| (NM_001131009)-SGPP1 | 7-14 |
| (NM_001131009)-UBR1 | 7-15 |
| (NM_001136127)-LYST | 1-7 |
| (NM_001143687)-CEP350 | 1-1 |
| CLK1-(NM_001131009) | 2-7 |
| RNF150-(NM_001131009) | 4-7 |
| TMEM30A-(NM_001131008) | 7-7 |
| ZRANB2-(NM_001131008) | 1-7 |

^*^These fusion genes were mostly identified by tools for the annotation and prediction of biologically functional gene fusion candidates. Although they may potentially serve a critical role in tumor pathogenesis and progression, their clinical significance cannot be determined at present and need to be further investigated.
